# Effectiveness of Low to Moderate Physical Exercise Training on the Level of Low-Density Lipoproteins: A Systematic Review

**DOI:** 10.1155/2018/5982980

**Published:** 2018-11-01

**Authors:** Ali M. Albarrati, Mansour Saleh M. Alghamdi, Rakan I. Nazer, Maarab M. Alkorashy, Nora Alshowier, Nichola Gale

**Affiliations:** ^1^Rehabilitation Health Sciences Department, College of Applied Medical Sciences, King Saud University, Riyadh, Saudi Arabia; ^2^Cardiac Sciences Department, College of Medicine, King Saud University, Riyadh, Saudi Arabia; ^3^King Faisal Specialist Hospital and Research Center, Riyadh, Saudi Arabia; ^4^Alhada Military Hospital, Taif, Saudi Arabia; ^5^School of Healthcare Sciences, Cardiff University, UK

## Abstract

**Background:**

Regular exercise reduces risk factors associated with cardiovascular disease (CVD). Elevated low-density lipoprotein (LDL) contributes to atherosclerosis formation, which is associated with an increased risk of CVD. The relationship between exercise therapy and lipid levels has been widely studied, but it is established that high-intensity exercise improves lipid profile. However, the effectiveness of low- to moderate-intensity exercise in altering LDL levels is controversial. This review aims to identify the current evidence and existing gaps in literature in this area.

**Methods:**

We searched and reviewed various randomized controlled clinical trials in the electronic databases EMBASE, CINAHL, the Web of Science, Cochrane, Pedro, Medline (PubMed), and Google Scholar using the keywords “low and moderate aerobic training,” “exercise”, “low-density lipoproteins,” “cholesterol,” “atherosclerosis,” and “coronary artery diseases markers.” We included studies that involved low- and/or moderate-intensity exercise training in apparently healthy adults over a period of 8 weeks and its effect on LDL levels. We selected a total of 11 studies from 469; nine were randomized controlled trials and two were systematic reviews.

**Results:**

Aerobic exercise of both low and moderate intensity resulted in a significant reduction of total cholesterol. Effects on low-density lipoprotein levels were significant, and most of the studies showed changes in the level without significant relation to the type of exercise. At the same time, exercise improved the health status and physical fitness of all the participants in the included studies.

**Conclusion:**

This study found that low- and moderate-intensity exercise and low-density lipoprotein levels were not proven to be significantly related, except in a few studies that were limited to dyslipidemia population.

## 1. Introduction

Cardiovascular disease (CVD) has increasingly become a global health problem and is a primary cause of morbidity and premature death worldwide [1]. A number of risk factors contribute to increased risk of CVD, including hyperlipidemia, aging, hypertension, and diabetes [2].

Hyperlipidemia is an increase in levels of circulating lipids in blood stream and considered a major contributing risk factor for the development of atherosclerosis that leads to CVD [3]. Atherosclerosis is a major cause of CVD, which occurs as a result of fatty deposition in the wall of the artery and, ultimately, plaque formation. Increased lipidemia, and in particular LDL, is associated with increased risk of CVD including coronary artery disease (CAD) and stroke [4].

Lack of regular exercise is a major cause of CVD and contributes to the pathogenesis of cardiovascular system disease via several mechanisms including atherosclerosis, which can be altered by physical activity. Physical activity has been defined as “any bodily movement produced by skeletal muscles that results in energy expenditure”. Exercise is a subset of physical activity which has been defined as a “planned, structured and repetitive bodily movement done to maintain or improve one or more components of physical fitness” (American College of Sports Medicine (ACSM), 2013) [5].

A number of observational studies show that a reduction in LDL levels lowers the risk of CVD and vice versa. Regular exercise is considered as an important part of CVD prevention and health optimization and plays a key factor in longevity [6]. Low- to moderate-intensity exercise uses lipid as a source of fuel during exercise and consequently improves the work capacity of the skeletal muscles, increases blood supply to different parts of the body, enhances vessels' ability to respond in demand to conduct blood efficiently, and at the same time reduces the peripheral vascular resistance to the blood. Previous literature has reported inconsistency regarding the effects of exercise on lipid metabolism, mainly due to the variations of mode and intensity of exercise employed. Therefore, it is necessary to conduct a robust review that aims to examine the effects of exercise programs on LDL levels. The purpose of this systemic review was to critically analyze the effectiveness of low- to moderate-intensity exercise training on LDL levels.

## 2. Methods

### 2.1. Data Sources

Following recent PRISMA recommendation, studies were retrieved using electronic databases including Medline (PubMed), CINAHL, EMBASE, Google Scholar, the Web of Science, the Cochrane Central Register of Controlled Trials, Pedro, and Dissertation Abstracts International between 2000 and October 2016. The following terms were used as search keywords (including MeSH terms): “low and the moderate-intensity aerobic training,” “exercise”, “low-density lipoproteins (LDL),” “cholesterol,” “atherosclerosis,” and “coronary artery diseases markers.” These terms were used in various combinations, such as “low-intensity aerobic training and/or moderate aerobic training and/or (low-density lipoproteins and/or cholesterol and/or coronary artery diseases markers).”

### 2.2. Study Selection

The inclusion criteria for this study were systematic reviews and randomized controlled trials that fulfilled the inclusion criteria such as (a) a period of > 8 weeks of aerobic training a lone, (b) population aged ≥ 18 years free from CVD/other pathology, and any form of statins (c) studies that were published in English between 2000 and October 2017, and (d) LDL levels measured while fasting before and after the entire period of training. Light to moderate exercise was defined as 50-74% of maximum heart rate, <60% of heart rate reserve, or VO_2_ max for 5 days a week. Studies were excluded if participants changed their diets and received any medical treatment for their elevated levels of LDL or cholesterol, or when subjects suffered from metabolic disorders, inflammatory diseases, diabetes, hypertension, or cardiorespiratory problems.

### 2.3. Data Extraction

Qualified studies were reviewed by two investigators using a standardized data collection form. This study used a matrix system to obtain studies and articles that had been reviewed. For this study, a coding/data sheet (Table 1) was used to measure assess the quality of the included studies. Each paper was score out of a maximum of 22 points for the best quality paper. The critical appraisal sheets and levels of evidence were obtained from the Centre for Evidence-Based Medicine (Table 2) [7, 8].

The critical appraisal sheet developed by Moher et al. 1999 was used for systematic reviews that were included in this paper [19]. It classifies studies according to the questions they ask; this is a beneficial approach in this systematic review, which aims to measure the effectiveness of interventions.

Data was extracted using an extraction sheet that allowed us to summarize the main items including author, year, design, main question, subjects, outcomes, conclusion, and evidence strength (Table 3).

## 3. Results

**Figure 1:**
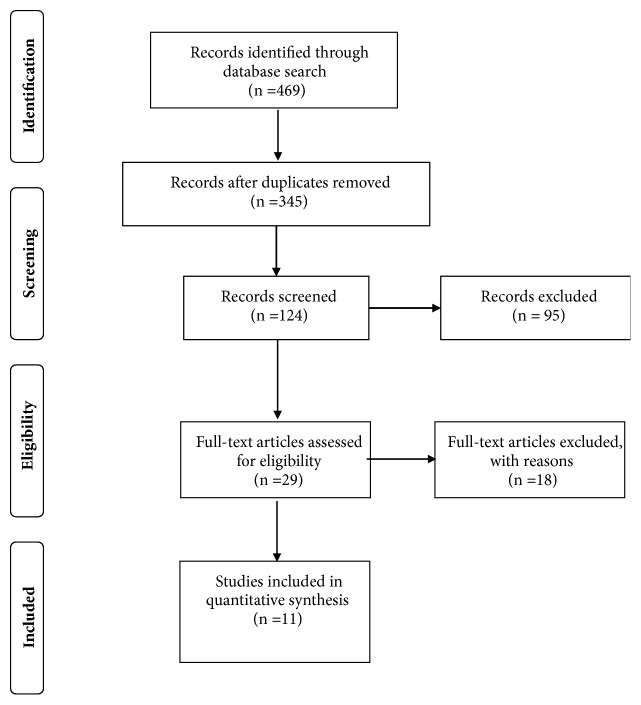
A flow of data selection.

The search of all databases revealed a total of 469 citations. The results were then exported to the reference manager Endnote X7 and duplications were removed, 324 studies, leaving 124 citations for examination. A total of 95 studies were excluded based on article, titles, and abstracts leaving 29 articles to be retrieved and for the full text to be appraised by both reviewers (AA and MA), independently (Figure 1). Differences of opinion were resolved by consensus between the two reviewers. Of the remaining citations, 18 full text articles were excluded for not meeting the inclusion criterion that the study is on cholesterol medications. The full selection process ultimately yielded 14 articles for inclusion in the present analysis.

Eleven studies met the inclusion criteria for this systematic review: nine randomized controlled trials and two systematic reviews. After the critical appraisal, seven RCTs scored level two with high scores ranging from 21 to 16, and two studies are with moderate scores ranging from 15 to 14. At the same time, two systematic reviews were divided after critical appraisal to level 1: one with a high score between 18 and 16 and another with a moderate score of 15 (Table 4).

There were 782 participants in this study pooled from all the studies (468 were females and 294 were males) between the age range of 18 and 75 years. The majority of the participants were of Caucasian ethnicity except for some studies that included black and Asian populations. A few studies did not mention the ethnicity of their subjects. The physical conditions of the included participants ranged from that of sedentary older individuals to athletes, but the majority of participants had lower physical activity profiles according the ACSM [5]. The participants' LDL levels were either normal or high borderline in nearly all of the studies, except one study, which included subjects with mild dyslipidemia. The majority of the studies included low and/or moderate supervised exercise, except for two studies, which utilized self-reported intensity of exercise. The exercise programs all included 30-45 min of aerobic exercises that included warm-up and cool-down phases 3-5 times a week for a period ranging from 8-24 weeks. Most of the studies did not include a follow-up for any changes in the LDL or other lipid levels after the intervention.

In 2010, a study by Yoshida et al. [9] examined the effect of moderate- to high-intensity aerobic exercise in a group of participants who suffered from moderate dyslipidemia. They demonstrated that exercise reduced LDL levels significantly* p*<0.01 and showed no difference between moderate-intensity and high-intensity exercise programs on the levels of LDL. However, this result may have come from the reduction of body weight, which was not treated as a confounding factor. Afzalpour et al. [10] showed no effects of exercise on LDL levels. However, this study was only on male subjects and the lack of significance may be attributed to the small sample size, which may have underestimated the influence of exercise on LDL levels. Additionally, the intensity of the training program is not enough to target LDL levels. In 2007, Halverstadt et al. [11] reported that a moderate-intensity endurance exercise program for 14 weeks reduced LDL levels when compared to the control subjects. Nevertheless, these results may have had a bearing effect of controlling the diet and fat during the study. Additionally, these findings may contribute to the positive effects of obesity management and physical activity.

Sittiwicheanwong et al. [12] examined the effects of moderate-intensity aerobic exercise in a group of sedentary Thai women. This study showed no significant reduction in the levels of LDL. However, the low sample size and huge dropouts from the control group (5 out of 20 subjects) may have underestimated the effect of the exercise. Additionally, the authors did not take into consideration the effects of other confounding factors, such as age variation and the menstrual cycle [12]. As part of the STRIDDE study, Slentz et al. found that light- and moderate-intensity exercise groups had a reduction in very low-density lipoproteins (VLDL). The low-intensity exercise group maintained the reduction for 15 days after detraining. Similarly, the moderate exercise group maintained their LDL levels after detraining, but for a longer period of time. This reduction was less pronounced in the nonactive group [13].

Murphy et al. evaluated the effects of worksite walking for 45 minutes twice a week at moderate intensity [14]. This failed to prove that walking can reduce the levels of LDL. However, this study had several limitations that would have contributed to insignificant results. There was a lack of power calculation to detect the significance of the walking program on the levels of LDL and inappropriate randomization, which could have contributed to selection bias and heterogeneity of the groups. There was also subjective classification of whether participants were active or sedentary and a lack of control of physical activities of the control group during the study. Additionally, the walking program was below the recommended dose suggested by the ACSM [5].

A study by Nieman et al. [15] showed no significant effect of moderate- to high-intensity exercise group p>0.05 on LDL level. Significant changes in LDL were observed in the exercise group with dietary modifications compared to exercise alone. Thus, it was recommended to combine exercise with lifestyle modifications to achieve favorable effects [15]. Kin Isler et al. [16] failed to find significant effects of moderate-intensity exercise on LDL levels. This may have been a result of several factors, such as a small sample size and lack of documentation of participants' diets. Though all the participants of this study were female college students, the influence of the menstrual cycle, which has an effect on lipoprotein metabolism, was not considered. Kraus et al. [17] examined the effect of increasing the volume and intensity of aerobic exercise upon the lipid profiles of 111 sedentary overweight participants diagnosed with mild to moderate dyslipidaemia. Participants were randomized to either 6 months in a control group or 8 months in one of three aerobic exercise groups. The three aerobic exercise groups included two groups with high-intensity and different volume exercises and one group on moderate-intensity/ low-volume exercise (walking for the calorific equivalent of 12 miles/week at an intensity of 40–55% V O_2_ peak). They reported that the moderate-intensity/low-volume exercise had no significant effect on the total plasma LDL, but it had important effects on the concentrations of LDL subfractions. A systematic review included a program that is more than 12 weeks [18].

Some confounding factors were not considered in these studies that might have affected the results, as they had not been mentioned, such as losing weight during participation in the study, menstrual cycle changes for female participants, and lifestyle modifications like cessation of smoking.

## 4. Discussion

This review included 11 RCTs that examined the effects of low- to moderate-intensity exercise on LDL levels. The review showed that the included studies had inconsistent results on the effects of exercise on LDL levels in the blood after only low- and moderate-intensity exercise. The majority of the studies could not identify significant changes in LDL levels with low-intensity exercise except for Slentz et al. [13] in which the researchers suggested beneficial effects of low-intensity exercise in comparison with different intensities, and the participants were able to maintain changes in LDL for a longer period. However, those changes could be due to many other factors like losing weight and the reduction of total body fat [20] and could be accompanied by restrictions of diet to maintain a specific weight [21]. Hence, low-intensity exercise in isolation cannot be used to induce therapeutic changes in the LDL levels, but its effects cannot be ignored, as keeping active is associated with improvements in other health indicators like general fitness, maximum oxygen consumption, body composition, physical activity, and blood pressure [13]. Low to moderate intensity exercise may be preferable and more sustainable than higher intensity exercise with additional effects on overall health.

Moderate-intensity exercises were the most commonly used type of exercise in the above-mentioned studies included in this review. Afzalpour et al. [10], Sittiwicheanwong et al. [12], Slentz et al. [13], Murphy et al. [14], Nieman et al. [15], and Kin Isler et al. [16] all found a significant reduction of LDL subfractions, but they were not able to significantly relate the reduction in LDL to the exercises [5, 10–15]. Similarly, a number of studies have examined the effect of moderate-intensity aerobic exercise on LDL particles but the results of these studies are controversial [22–24]. Varady and colleagues [22] reported that aerobic exercise after a few months decreased the concentration of atherogenic small LDL subfractions and increased the average size of LDL particles in patients with hypercholesterolemia. In contrast, Elosua et al. examined the effect of aerobic exercise on sedentary healthy individuals and found that aerobic exercise had no effect on LDL particle diameter [23].

However, the majority of them recommend considering general health benefits compared to other lipoproteins in the blood among several populations ranging between young and old sedentary subjects who had different health problems like obesity and mild to moderate hyperlipidemia. The findings of these studies are in consistent with previous studies, which suggested that moderate-intensity exercise had nonsignificant lowering effects on LDL [8]. On the other hand, moderate-intensity exercise has a significant effect on the LDL levels in mild to moderate hyperlipidemia [9] and among middle-aged sedentary healthy subjects [11]. In fact, this also had previously supported from a meta-analysis [8], which is similar to the present analysis that could not find a significant relationship between reductions of LDL levels with exercise. Previous studies included exercise programs of moderate intensity that extended for longer periods relative to other included studies, and exercise programs utilized for those studies were under extensive supervision of exercise specialists; this could be one of the reasons that caused exercise to have a significant influence on the levels of LDL. Thus, Kraus et al. recommended a moderate-intensity exercise regimen to induce changes in the blood's lipoproteins in general and LDL particles as one of them, which included 12 miles/week of moderate jogging [21].

Many confounding factors were not controlled during the studies, like smoking, diet, weight loss, total fat loss, adherence to exercise, and direct supervision of an exercise specialist. Part of the confounding factors were shown to influence different lipoproteins, especially LDL [20, 25–29], and the majority of them existed in the subjects of the included studies. Furthermore, the included studies investigated different treatments for elevated LDL among different subjects. The subjects in the included studies were from different socioeconomic, cultural, and educational backgrounds. Thus, results may vary according to the different associated factors, and that may have influenced the body's responses to abnormal levels and affinity of LDL particles to their arteries and forming plaque [20]. However, some of the included studies did not detail the demographics of the participants, so it will be difficult to examine the extent of that factors in those studies.

## 5. Limitations

This study was limited by the timeline of the included studies and included only those published in English. This might have limited the number of the included studies. Another limitation was exclusion of other modalities of exercises like resistance exercise with moderate aerobic exercise. In this review, we only focus on plasma LDL and we did not include its subfractions which impose a risk to cardiovascular system and may be reduced by aerobic exercise. Despite these limitations, this study provided an up-to-date perspective on the current evidence about low- to moderate-intensity exercise and changes in LDL levels.

## 6. Conclusion

This study found that low- to moderate-aerobic exercise intensities did not reduce the levels of LDL except in few studies that have been limited to specific populations. Thus, the effects of low- and moderate-intensity exercise on LDL level should not be ignored as low- to moderate-aerobic exercise intensities have shown a positive effect on LDL subfractions.

Frequent moderate-intensity aerobic exercise should be recommended for sedentary subjects to avoid risks associated with dyslipidaemia. Regular moderate aerobic exercise is link to reducing the risks of cardiovascular disease.

## Figures and Tables

**Table 1:** Coding sheet for critical appraisal.

Quality components	Yes/No (1/0)	Factors of the quality
Background and aim		a thorough research of related literature has been done and described well in the background of the study
		illustrated significance of the study has been mentioned
		clear aim of the study has been included

Design		number of the groups in the study
		blinding of the study
		baseline for the targeted measurement has been developed
		sample size was appropriate to answer the question of the study

Subjects		recruitment of subjects was illustrated clearly and following a strategy that is appropriate for the design
		the inclusion and exclusion criteria of the subjects stated clearly
		Adherence of the patients to the suggested protocol
		withdrawal percentage of subjects from the sample size before statistical analysis does not affect the statistical analysis of the study
		groups in the study were from similar environments

Intervention		treatment protocol was described clearly
		treatment is equal for all subjects
		treatment efficacy was measured precisely

Outcome		the outcome of the study was illustrated clearly
		supported for clinical importance with validity and reliability
		follow up period to measure the persistence of the effects after the intervention

Analysis		statistical analysis is well illustrated and suitable to measure the efficiency of the treatment
		the statistical analysis has been supported by mention of the significance of the intervention (*p*-value)

The study is replicable		The study is replicable by other researchers because of availability of the previous factors

Recommendation and conclusion		Recommendation and conclusion of the study were stated clearly and related to the results of the study

**Table 2:** Level of evidences.

Question studies asked	Level of evidence (Step)	Explanation of the level
Does the intervention help?	Level 1	Systematic review of randomized trials or *n*-of-1 trials
	Level 2	Randomized trial or observational study with dramatic effect
	Level 3	Non-randomized controlled cohort/follow-up Study
	Level 4	Case-series, case-control studies, or historically controlled studies
	Level 5	Mechanism-based reasoning

**Table 3:** Data extraction.

Author/year	Topic	Design	Sample	Type of exercise	Critique	Conclusion	Level of evidence
Yoshida et al. (2010) [9]	The purpose was to examine the effect of a supervised aerobic training program for 16 weeks in purpose to improve the lipid metabolism in dyslipidemic, moderately obese Japanese patients.	RCT	N=25 (males=22, females=3) dyslipidemic patients with mean age of 39 years did not have metabolic disorders, heart problems, sever obesity and did not use medications that affect the blood lipids.	Supervised aerobic exercises of walking, cycling or swimming, for 60 min/ 2-3 times/ 16 weeks at 60-80% MHR each session started with 10 min warming up and finished with 10 min of cooling down.	LDL had decreased significantly from 147±27 to 131±21 by week 8 and decreased to 129±21 by week 16 *p*<0.001.	Supervised aerobic exercises 2-3 times a week with weight reduction had favorable effects on lipid profiles and insulin sensitivity.	Level 2 (17/22)

Afzalpour et al. (2008) [10]	The purpose was to measure the effects of different intensity exercises on blood lipids and serum oxidized LDL.	RCT	N=45 (males between age between 29-37) healthy, with no confounding factors might affect the subjects lipid profile randomly assigned to: MAE n=17 VAE n=15 Control group n=12	Moderate intensity= 30-45 min of brisk walking and stepping at 60-65% MHR for 8 weeks. High intensity exercise= the same program with running at 80-85% MHR. Each session included 10-15 min warming up and ended with 5-10 min cooling down.	No reduction of LDL has been noticed among all groups except the exercise groups did not deteriorate like the control group.	No reduction of the serum oxidized LDL due to exercises program.	Level 2 (16/22)

Halverstadt et al, (2007) [11]	Measured the independent effects of 24 weeks standardized aerobic exercise program on the plasma lipoproteins and their subfractions	RCT	N=100 (females=58, males=42) at the age of 58±0.6, healthy, sedentary with no more than 20 min activities twice a week, without metabolic disorders, and habits might affect the subject lipid profile. Mean LDL was 129±2.7 mg/dl at the baseline.	Different aerobic exercises including: bikes, treadmills, elliptical machines, skier machines, stepping machines, and rowers, for 24 weeks began with 20 min 3 times a week at 50% VO2max for 10 weeks, the following 14 weeks exercise program was 40 min 3 times/week, at 70% of VO2max	This exercise program has a significant reduction effect on LDL, at the end of exercise LDL new reading was (-0,7±1.7/dl) *p*<0.0001.	Significant favorable effects on the concentration and particles size of the blood lipids, improvement of the physical fitness and maximum oxygen consumption of the subjects	Level 2(20/22)

Sittiwicheanwong et al. (2007) [12]	Examined the effect of aerobic exercises on the LDL and other serum fatty lipids among middle age sedentary women.	RCT	n=40 females at the age of 40-55 years 14 of them were menopausal (all healthy, sedentary, Thai women and did not use any lipid management medications). 20 subjects voluntarily joined the exercise program. 20 subjects assigned to the control group to match the results, 5 subjects from the control group excluded due to drop out and increasing their daily activities. Statistical analysis has been done on the remaining 35 subjects.	(EG)10 min of warming up, followed by 25 min of ergometer cycling followed by 10 min cooling down at 60% of MHR, 3 days/ week/ 12 weeks. (CG) to match the results	LDL did not change but other subfractions of LDL had changed	Moderate intensity exercises have a favorable effect to prevent subfractions of LDL to be more atherogenic and that will help to prevent cardiovascular diseases.	Level 2 (14/22)

Slentz et al. (2007) [13]	Measured the effects of exercises on the lipid profile after the cessation of the exercises, and how long that effect can be maintained	RCT	N=240 (females=130, males=110 two of the high intensity and two of the moderate intensity exercise groups had been excluded from the statistical analysis because of their lipid profile were higher than the standard deviation of the means in all groups but that did not affect the final results) sedentary, overweight, at the age of 45-60 years, and dyslipidemic where LDL= 130-190. Those subjects were randomly assigned to one control group or other three exercises groups.	Three exercise groups: Low amount, moderate intensity: walking 12 miles/week at 40-55% of VO2max for 6 months. Moderate amount, vigorous intensity= brisk walking or running 12 miles/ week at 65-80% of VO2max for 6 months. High amount, vigorous intensity= brisk walking or running 20 miles/ week at 65-80% of VO2max for 6 months	The inactivity of the control group deteriorated their LDL level than baseline. The moderate and high intensity exercise group had not maintained the reduction of the LDL during the detraining period. the low intensity exercise group had a significant reduction in the LDL and that reduction had been maintained after five days of the last training session but not after 15 days, *p*<0.01. LDL=(-3.3mg/dl) in 22 subjects.	The study results suggested that effects of different exercises were limited to 2 weeks after the detraining, and tailored exercise programs must be prescribed individually and VLDL control was more related to the low intensity exercises.	Level 2 (21/22)

Murphy et al. (2006) [14]	Evaluation of the worksite walking among sedentary workers	RCT	N=37 (females=24, males=13) at age of 41.5±9.3 workers from Ireland without any major health problem that might affect the cardiovascular diseases markers, did not use any medications that change their lipid profiles, and women who were pregnant or planning to get pregnant in the next 5 months after the beginning of the study. Randomly assigned to either of two groups walking and control group in a ration 3 walkers:2 control.	Walking program with self monitoring of speed started at 25 min first week, progressed to 35 min second week and the next 6 weeks 45 min twice a week at nearly 62% of MHR.	The suggested walking program failed to induce an effect on the LDL levels among the exercise group *p*>0.05.	No significant effects of the program on the cardiovascular diseases markers *p*>0.05. However it was able to improve the general fitness, body fats, and systolic blood pressure of the participants.	Level 2 (15/22)

Nieman et al. (2002) [5]	Measured the responses of different serum lipids to exercises among moderately obese women in with dietary intervention and without	RCT	N=91 (females), at the age of 25-75 years. All subjects were sedentary, moderately obese without medical problems, did not have factors might alter their lipid profiles, and did not follow diet programs. All subjects were randomly assigned into each of four groups (control, exercise, diet, and exercise and diet)	Exercise program includes 45 min walking/ 5 times/ week/ 12 weeks at 60-80% MHR. The first 3 weeks of exercise program start at 20-25min walking 5 times/ week at 60-65% MHR, the next weeks continued the standard program at intensity of 70-80% MHR.	No significant change of the LDL value *p>0.05 *in the exercise group only after 12 weeks of the exercise intervention.	Since there was no weight reduction in the exercise group, the study concluded that favorable changes of the lipid profiles were in conjunction with diet modification and exercise with diet modification intervention.	Level 2 (19/22)

Kin Isler et al. (2001) [15]	The purpose was to examine the effect the regular aerobic dancing and the stepping aerobic dancing on lipid profiles of sedentary college age females.	RCT	N=45 females college students) divided into three groups. Step aerobic dancing group n=15, regular aerobic dancing n=15 and control group n=15.	Aerobic dancing and step aerobic dancing for 45 min/3 days/8 weeks with moderate intensity of 60-70% MHR. Each session includes starting with 5-7 min warming up then rhythmic movements like jumping and stretching 20 min, then 10 min of floor exercises for abdomen and lower limbs, finished with 5 min cooling down.	No significance change in LDL in all three groups *p*>0.05.	Aerobic dancing is an effective method to modify Total cholesterol and Total cholesterol: HDL in college age female students but no LDL.	Level 2 (16/22)

Kraus et al. (2002) [16]	This study investigated the effect of the amount and intensity of exercise on the plasma lipoprotein in the population of overweight, obese, and mild to moderate hyperlipidemic males and females.	RCT	N=159 between 40-45 years old, sedentary, overweight, or mildly obese. With LDL=130-190 mg/dl or HDL= males<40 and females <45 mg/dl. By the end of the study 47.2% of the subjects dropped out due to different reasons and that left 84 subjects for the statistical analysis. Subjects were randomly assigned into four groups (control group, high amount-high intensity, low amount-high intensity, and low amount-moderate intensity). Weight control methods had been applied to control loss of the weight and eliminate that as a confounding factor, any change in the body weight by more than 5% than the baseline weight excluded from the analysis.	High amount-high intensity= jogging 20 mi/week at 65-80% of VO2 max. Low amount-high intensity= jogging 12 mi/week at 65-80% of VO2 max. Low amount-moderate intensity= 12 mi/week at 40-55% VO2 max. The exercises included treadmill, elliptical trainer, and cycling ergometer. The six months exercise training period preceded by a two to three months period of graded training.	No significant changes on the concentration of plasma LDL at the end of the study. In the same time, there were significant changes of the LDL subfractions and LDL particles size, and those changes were directly proportional to the increase of exercises level.	The higher the exercise's intensity, the higher the benefits on the general body fitness. However, low intensity exercises will elect benefits to the body but higher intensity exercises were more beneficial. Improvement in the blood lipid profile will be gained by performing a moderate pace jogging for 17 to 18 mi/week.	Level 2 (15/22)

Asikainen et al. (2004) [17]	A systematic review for randomised, controlled exercise trials on postmenopausal women on components of health related fitness including metabolic profile	Systematic Review	Postmenopausal women between 55-65 years old, sedentary, overweight, or mildly obese <33 BMI.	Walking or walk-jogging for 30 to 60 minutes 2–5 days/week at intensity of 40–75% of VO_2_max for 10 weeks to 1 year.	No significance change in LDL in all exercise studies alone except for those combined with weight reduction management.	Moderate intensity exercise alone has no effect on the levels of LDL. While moderate exercise intensity combined with weight reduction management shows a significant decrease in the levels of LDL.	High Level (16/22)

Tambalis et al. (2009) [18]	The effectiveness of aerobic exercise training with different intensities (moderate and high) as well as the type of exercise (aerobic, resistance, and combined aerobic with resistance) in altering the blood lipids.	Systematic Review	Included RCT and case control studies between 1991 to 2006 with exercise program of no less than 12 weeks, (2) sedentary, apparently healthy adult individuals aged between 18 and 84 years, (3) both hyperlipidemic and normolipidemic adults, and (4) assessments of at least one of the lipids (TC, HDL-C, LDL-C, and TG) in the fasting state.	Walking, treadmill walking, jogging, or cycle ergometer, aerobic activities and swimming. The duration of the training program ranged from 12 weeks to 24 months and the frequency of training varied from 2 to 7 sessions per week.	Moderate intensity did not reduce the levels of plasma LDL.	Moderate intensity exercise has no favorable influence on the levels of LDL independently of the total volume of exercise.	Moderate Level (15/22)

**Table 4:** Final scores of all studies.

Study	Yoshida et al. (2010)	Tambalis et al. (2009)	Afzalpour et al. (2008)	Halverstadt et al, (2007)	Slentz et al. (2007)	Sittiwicheanwong et al. (2007)	Murphy et al. (2006)	Asikainen et al. (2004)	Nieman et al. (2002)	Kraus et al. (2002)	Kin Isler et al. (2001)
Background and aim	3/3	2/3	3/3	3/3	3/3	2/3	2/3	3/3	3/3	3/3	2/3

Design	1/4	2/4	1/4	4/4	4/4	2/4	2/4	3/4	3/4	2/4	2/4

Subjects	4/5	4/5	5/5	5/5	4/5	2/5	5/5	2/5	5/5	2/5	5/5

Intervention	3/3	2/3	3/3	3/3	3/3	3/3	1/3	3/3	3/3	3/3	3/3

Outcome	2/3	1/3	1/3	1/3	2/3	1/3	1/3	1/3	1/3	1/3	1/3

Analysis	2/2	2/2	1/2	2/2	2/2	2/2	2/2	2/2	2/2	2/2	1/2

The study is replicable	1/1	1/1	1/1	1/1	1/1	1/1	1/1	1/1	1/1	1/1	1/1

Recommendation and conclusion	1/1	1/1	1/1	1/1	1/1	1/1	1/1	1/1	1/1	1/1	1/1

Total score	17/22	15/22	16/22	20/22	21/22	14/22	15/22	16/22	19/22	15/22	16/22

## Abbreviations

CAD: Coronary artery disease
LDL: Low-density lipoproteins
HDL: High density lipoproteins
RCT: Randomized control trial
CVD: Cardiovascular disease.

## References

[1] Salomon J. A., Wang H., Freeman M. K. (2012). Disability-adjusted life years (DALYs) for 291 diseases and injuries in 21 regions, 1990-2010: a systematic analysis for the Global Burden of Disease Study 2010. *Lancet*.

[2] D'Agostino R. B., Vasan R. S., Pencina M. J. (2008). General cardiovascular risk profile for use in primary care: the Framingham heart study. *Circulation*.

[3] Ross R. (1999). Atherosclerosis--an inflammatory disease. *The New England Journal of Medicine*.

[4] Brunzell J. D., Davidson M., Furberg C. D. (2008). Lipoprotein management in patients with cardiometabolic risk: consensus conference report from the american diabetes association and the american college of cardiology foundation. *Journal of the American College of Cardiology*.

[5] Tharrett S. J., Peterson J. A. (2011). *American College of Sports Medicine., ACSM's Health/Fitness Facility Standards And Guidelines*.

[6] Eto Y., Koike A., Matsumoto A. (2004). Early aerobic training increases end-tidal CO2 pressure during exercise in patients after acute myocardial infarction. *Circulation Journal*.

[7] Philip B. C. B., Sackett D., Badenoch D., Strauss S., Haynes B., Dawes M. (2005). *Centre for Evidence Based Medicine Levels of Evidence*.

[8] Halbert J. A., Silagy C. A., Finucane P., Withers R. T., Hamdorf P. A. (1999). Exercise training and blood lipids in hyperlipidemic and normolipidemic adults: A meta-analysis of randomized, controlled trials. *European Journal of Clinical Nutrition*.

[9] Yoshida H., Ishikawa T., Suto M. (2010). Effects of supervised aerobic exercise training on serum adiponectin and parameters of lipid and glucose metabolism in subjects with moderate dyslipidemia. *Journal of Atherosclerosis and Thrombosis*.

[10] Afzalpour M. E., Gharakhanlou R., Gaeini A. A., Mohebbi H., Hedayati M., Khazaei M. (2008). The effects of aerobic exercises on the serum oxidized LDL and total antioxidant capacity in non-active men. *CVD Prevention and Control*.

[11] Halverstadt A., Phares D. A., Wilund K. R., Goldberg A. P., Hagberg J. M. (2007). Endurance exercise training raises high-density lipoprotein cholesterol and lowers small low-density lipoprotein and very low-density lipoprotein independent of body fat phenotypes in older men and women. *Metabolism - Clinical and Experimental*.

[12] Sittiwicheanwong R., Ariyapitipun T., Gulsatitporn S., Nopponpunth V., Abeywardena M., Dahlan W. (2007). Alterations of atherogenic low-density lipoproteins and serum fatty acids after 12 week moderate exercise training in sedentary Thai women. *Asia Pacific Journal of Clinical Nutrition*.

[13] Slentz C. A., Houmard J. A., Johnson J. L. (2007). Inactivity, exercise training and detraining, and plasma lipoproteins. STRRIDE: a randomized, controlled study of exercise intensity and amount. *Journal of Applied Physiology*.

[14] Murphy M. H., Murtagh E. M., Boreham C. A. G., Hare L. G., Nevill A. M. (2006). The effect of a worksite based walking programme on cardiovascular risk in previously sedentary civil servants. *BMC Public Health*.

[15] Nieman D. C., Brock D. W., Butterworth D., Utter A. C., Nieman C. C. (2002). Reducing Diet and/or Exercise Training Decreases the Lipid and Lipoprotein Risk Factors of Moderately Obese Women. *Journal of the American College of Nutrition*.

[16] Kin Isler A., Kosar S. N., Korkusuz F. (2001). Effects of step aerobics and aerobic dancing on serum lipids and lipoproteins. *The Journal of Sports Medicine and Physical Fitness*.

[17] Kraus W. E., Houmard J. A., Duscha B. D. (2002). Effects of the amount and intensity of exercise on plasma lipoproteins. *The New England Journal of Medicine*.

[18] Tambalis K., Panagiotakos D. B., Kavouras S. A., Sidossis L. S. (2009). Responses of blood lipids to aerobic, resistance, and combined aerobic with resistance exercise training: a systematic review of current evidence. *Angiology*.

[19] Moher D., Cook D. J., Eastwood S., Olkin I., Rennie D., Stroup D. F. (1999). Improving the quality of reports of meta-analyses of randomised controlled trials: the QUOROM statement. *The Lancet*.

[20] Szapary P. O., Bloedon L. T., Foster G. D. (2003). Physical activity and its effects on lipids. *Current Cardiology Reports*.

[21] (2002). Third report of the national cholesterol education program (ncep) expert panel on detection, evaluation, and treatment of high blood cholesterol in adults (adult treatment panel iii) final report. *Circulation*.

[22] Varady K. A., St-Pierre A. C., Lamarche B., Jones P. J. H. (2005). Effect of plant sterols and endurance training on LDL particle size and distribution in previously sedentary hypercholesterolemic adults. *European Journal of Clinical Nutrition*.

[23] Elosua R., Molina L., Fito M. (2003). Response of oxidative stress biomarkers to a 16-week aerobic physical activity program, and to acute physical activity, in healthy young men and women. *Atherosclerosis*.

[24] Wang Y., Xu D. (2017). Effects of aerobic exercise on lipids and lipoproteins. *Lipids in Health and Disease*.

[25] Hambrecht R., Gielen S., Linke A. (2000). Effects of exercise training on left ventricular function and peripheral resistance in patients with chronic heart failure: a randomized trial. *Journal of the American Medical Association*.

[26] Smith J. K., Dykes R., Douglas J. E., Krishnaswamy G., Berk S. (1999). Long-term exercise and atherogenic activity of blood mononuclear cells in persons at risk of developing ischemic heart disease. *The Journal of the American Medical Association*.

[27] Fattirolli F., Cellai T., Burgisser C. (2003). Physical activity and cardiovascular health: a close link. *Monaldi Archives for Chest Disease*.

[28] Shepherd J. (1999). Risk factor modification extends the benefit of coronary artery revascularisation procedures. *International Journal of Clinical Practice*.

[29] Castelli W. P., Griffin G. C. (1997). *Good fat, Bad Fat : How to Lower Your Cholesterol and Reduce the Odds of a Heart Attack*.

